# Impacts of Anthropogenic Disturbance on Vegetation Dynamics: A Case Study of Wadi Hagul, Eastern Desert, Egypt

**DOI:** 10.3390/plants10091906

**Published:** 2021-09-14

**Authors:** Ramadan Bedair, Amira A. Ibrahim, Amal A. Alyamani, Salman Aloufi, Samah Ramadan

**Affiliations:** 1 Department of Botany and Microbiology, Faculty of Science, Al-Azhar University, Nasr City, Cairo 11884, Egypt; RamadanIbrahim.211@azhar.edu.eg; 2 Department of the Plant Protection and Biomolecular Diagnosis, Arid Lands Cultivation Research Institute (ALCRI), City of Scientific Research and Technological Applications (SARTA, City), New Borg El Arab City 21934, Egypt; 3 Department of Biotechnology, Faculty of Sciences, Taif University, P.O. Box 11099, Taif 21944, Saudi Arabia; a.yamani@tu.edu.sa (A.A.A.); s.aloufi@tu.edu.sa (S.A.); 4 Botany Department, Faculty of Science, Mansoura University, Mansoura 35516, Egypt; samahmahmoud@mans.edu.eg

**Keywords:** plant diversity, anthropogenic impacts, vegetation dynamics, wildlife, Wadi Hagul, DCCA, SAVI

## Abstract

Irresponsible human interventions, encroachment of natural habitats, and climate change negatively affect wildlife. In this study, the effects of human influence on Wadi Hagul, an unprotected area in the north of the Egyptian Eastern Desert that has recently been subjected to blatant encroachments of vegetation, were studied. The most important of these threats is the construction of the new road Al-Galala–Wadi Hagul–Zafarana. In Wadi Hagul, 80 species are reported in this study; the most represented plant families are Asteraceae (15 species) and Brassicaceae (6 species). Perennial, chamaephyte and Saharo-Arabian species were recorded in the highest percentage. Detrended canonical correspondence analysis showed that latitude, longitude, altitude, silt, sand contents, pH, and CO_3_^2^^−^ content are the factors that have the highest effect on vegetation distribution in the studied stands. Several invasive and alien species such as *Euphorbia prostrata* have been listed; these species typically have a negative effect on native species. The Soil Adjusted Vegetation Index (SAVI) indicated a decrease in plant cover during the study period, as compared to previous years. In 2013 and 2020, SAVI ranged from −0.02 to 0.42 and from −0.18 to 0.28, respectively. Recently, the violation and destruction of wildlife have increased, therefore, preserving it along with general biodiversity has become an urgent necessity.

## 1. Introduction

Land degradation and vegetation reduction caused by external stresses, affect biodiversity and natural ecosystems and the numerous services they provide [1]. Environmental deterioration, habitat changes, inappropriate vegetation management, translocation, fragmentation, and deforestation modify biotic and abiotic ecosystem components, resulting in changes in ecological processes such as vegetation dynamics [2,3,4]. Overgrazing, road building, overharvesting, solid wastes, salinization, industrialization, urbanization, and military activities are considered to be the main anthropogenic activities that lead to changes and transformation of vegetation and natural habitat loss in arid and semi-arid environments [5,6,7].

In terrestrial ecosystems, climate and land cover changes such as cover, height, biomass, relative humidity, soil temperature, moisture, fertility, and erosion affect the structural properties of vegetation [8]. Biodiversity decline is influenced by various types of human activities, including land cover changes, the introduction of invasive species, overexploitation, and pollution [9,10,11]. Alteration of vegetation growth is the result of climate variation, and human activities can modify atmosphere–biosphere interaction, causing changes in the hydrological cycle, either directly or indirectly [12,13,14].

Environmental issues, including soil fertility decline, heavy winds, increase in the evaporation rate, high temperature, and heavy rainfall, lead to dramatic changes in plant species community structure [7,15,16]. Ecosystem services and biodiversity contribute approximately 57% of gross domestic production [17], therefore, there is a pressing need to save biodiversity, especially endangered species through convert these locations to protected areas [18]. There are a variety of approaches that are able to reverse biodiversity loss, ranging from economic, through ecological, to ethical [19].

Human pressures in Africa, include agricultural and pasture activities, illegal timber harvesting, and bush fires. All of these stresses have a negative impact on the plant ecosystem [20]. From the middle of the last century, the human population of the earth doubled [21]. Population growth and urbanization are the most important causes of ecosystems collapse [22]. Rapid population growth leads to many problems such as fire prevalence, air pollution, light pollution, loss of genetic diversity, the prevalence of invasive species, and wildlife destruction [23]. Human activities, through the civilizational and agricultural expansion and by the destruction of natural habitats, have increased extinction rates up to 500 times [24] (Baillie et al., 2004). Over the ages, human activities have caused three powerful waves of extinction [25]. It is estimated that nearly 8390 plant species are listed as endangered [26]. Approximately 32% of the existing plant species are classified as either critically endangered or extinct [27], nearly 20% being extinct because of human activities [28]. In China, nearly 11% of the plant species that were evaluated are extinct [29].

Species with a narrow distribution range are more likely to be lost, while widespread species are more likely to survive [30]. Some wild plants have adapted to the conditions and survived in their natural habitats [31]. However, invasive species spread causes disturbance in communities and may lead to the extinction of endemic and native species [32].

Anthropogenic activities are causing changes in natural plant communities in Egypt from ancient times through draining of lakes and marshes and reduction of species number in aquatic communities [33]. Remote sensing is one of the most unique techniques used for estimating environmental changes [34]. Some characteristics of vegetation detected using remote sensing techniques are photosynthetic activity, chlorophyll content, plant density, green leaf biomass, the leaf area index, and plant health [35]. The second factor that includes climatic measures includes air temperature and solar radiation [36,37,38]. SAVI is one of the most common vegetation indices used as a tool to discriminate vegetation covers [39]. SAVI can be used efficiently in arid regions [40]. In areas where vegetative cover is low (i.e., <40%) and the soil surface is exposed, the reflectance of light in the red and near-infrared spectra can influence vegetation index values. This is especially problematic when comparisons are being made across different soil types that may reflect different amounts of light in the red and near-infrared wavelengths (i.e., soils with different brightness values). The soil-adjusted vegetation index was developed as a modification of the Normalized Difference Vegetation Index (NDVI) to correct for the influence of soil brightness when vegetative cover is low [41]. The output of SAVI is a new image layer with values ranging from −1 to 1. The lower the value, the lower the amount/cover of green vegetation [42].

A wadi is a natural depression on the earth’s surface. Wadi Hagul is a morphotectonic depression located between Gebel Ataqa’s southern scarps in the north and El-Galala El-Bahariya Plateau’s northern scarp in the south. Wadi Hagul is one of the unprotected wadies in Egypt and is one of the most anticipated project areas in Egypt to alleviate congestion. As a result, several integrated development projects are being worked on that implement many critical infrastructure areas such as quarries, roads, power plants, mines, landfills, and resorts. The most important national project is the development of the northern part of the Gulf of Suez pipeline surcharge, implemented through the main track of the study area and its environment [43]. Three main sectors can be recognized based on the vegetation and geological aspects of Wadi Hagul: upstream, middle, and downstream. Two distinct plant communities can be found in the upstream section of Wadi Hagul’s main channel: one is dominated by *Zilla spinosa* on elevated terraces of mixed deposits. The other is dominated by *Launaea spinosa* and represents a further stage in the building up of the wadi bed. In the middle section, *Leptadenia pyrotechnica* community occurs. In the downstream section, the vegetation is dominated by *Hammada elegans* with individuals of *Launaea spinosa* and *Lygos raetam* [44].

This paper focuses on Wadi Hagul, which has neither attained sufficient interest from intended responsible parts nor ecological studies, although it is considered an environment rich in various plants including a number of endangered plants, whereas other Eastern desert wadis of Egypt have received both interest and studies convenient for them. Therefore, the objective of this study is the estimation of vegetation cover in Wadi Hagul through the illustration of the relationship between environmental factors in the studied localities and the distribution of plant communities. The study also aimed at comparing vegetation changes and at testing new methods to record the reasons for changes in vegetation dynamics by calculating the temporal patterns of SAVI during 2013, 2015, and 2020 in Wadi Hagul, Eastern Egyptian Desert.

## 2. Materials and Methods

### 2.1. Study Area

Wadi Hagul lies between 32°09′32″ E and 32°17′27″ E and between 29°48′28″ N and 29°57′43″ N, with an area of 350 km^2^ (0.16% of the Eastern Desert total area). The main canal of Wadi Hagul extends until it reaches the Gulf of Suez, with a width ranging from 6 to 10 km and a length of approximately 36 km. Wadi Hagul is considered to be one of 15 wadies located on the Red Sea coast. It is situated 112 km west of Cairo, between Gebel Ataqa and the Kahaliya ridge [44]. Wadi Hagul is characterized by a dry desert climate with little rain, high temperatures, and a high evaporation rate. The three sections represent dry natural habitats inhabited mainly by xerophytic plants. The dominant species are Fagonia mollis, Echinops spinosus, and other xerophytic plants [45].

### 2.2. Climate Data

Climate data for the past 30 years (1990–2020) were extracted from www.meteoblue.com; accessed on 15 April 2021. They include mean maximum temperature (°C), mean minimum temperature (°C), precipitation (mm), hot days (°C), and cold nights (°C) (T S1). Wind direction and velocity (km/h) are provided in Figure S2.

### 2.3. Floristic Study

**Figure 1:**
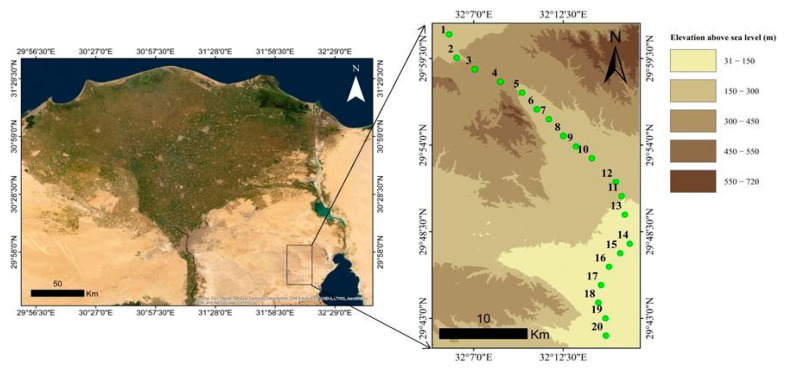
Location on the map showing the digital elevation model of Wadi Hagul.

**Figure 2:**
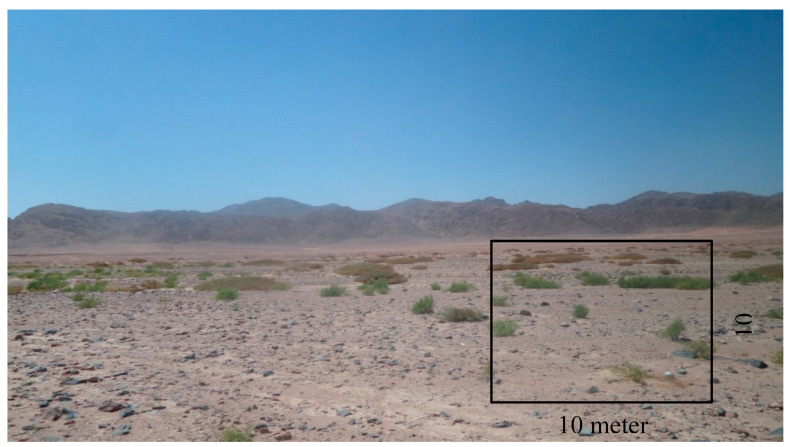
A quadrate with an area of 10 m × 10 m in.

To estimate the vegetation cover in Wadi Hagul, 20 stands were chosen to represent different plant habitats in Wadi Hagul. A total of 20 stands were selected for the study of the vegetation of Wadi Hagul during the spring and summer seasons of 2019 and 2020. In each stand, four quadrates were chosen (quadrate area = 10 m × 10 m = 100 m^2^) (Table 1 and Figure 1 and Figure 2). Vegetation cover was measured using visual estimation. Recorded plant species in all localities were identified, and life span was documented according to Boulos [46,47,48,49,50]. Life form and floristic category were recognized after Raunchier [51], Tutin et al. [52], and Davis [53].

### 2.4. Soil Analyses

Many soil factors were examined in order to determine the characteristics of the soil in the study area. From each stand, four soil samples were collected and merged to form a homogeneous and representative sample of a single locality. Fifteen soil factors were analyzed: soil texture (sand, silt, and clay), pH, total dissolved salts, electrical conductivity, magnesium, calcium, sodium, potassium, chloride, bicarbonate, carbonate, sulfate, and organic carbon content. Soil texture was determined according to Estefan et al. [54] using a pipette method. Digital portable pH, TDS, and EC meters (Adwa^®^, Adwa Instruments, Szeged, Hungry) were used to calculate pH, total dissolved salts (T.D.S.), and electrical conductivity (E.C.). Magnesium and calcium were determined using a titration method according to Page et al. [55]. A flame photometer at wavelengths of 589 and 767 nm was used to determine sodium and potassium, respectively [54]. Chlorides, bicarbonates, and carbonates were determined using a titration method [56]. Sulfates were determined after Estefan et al. [54] using the turbid metric method. Organic carbon was determined using a titration method [57].

### 2.5. Statistical Analyses

Descriptive statistical measures (mean, median, standard deviation, range, minimum, maximum, and interquartile range) for different soil factors were determined using Sigmaplot ver. 12.5.

Several biodiversity indicators were measured such as Shannon′s and Simpson′s diversity indices, species richness, and evenness were extracted using PC-ORD ver. 5 software [58]. Hills numbers were calculated using the R program.

To clarify the relationship between environmental factors, especially soil factors, the study area was divided into three areas: the beginning of the Wadi (stands 1 to 7), the middle (stands 8 to 14), and the end of the Wadi (stands 15 to 20). A detrended canonical correspondence analysis (DCCA) was performed using CANOCO ver. 4.5 and CanoDraw ver. 4.1 [59].

### 2.6. Vegetation Change

To confirm the negative impact of human interventions on the vegetation cover in Wadi Hagul, SAVI was calculated in the study area during different years. Satellite images for 2013, 2015, and 2020 were obtained from the Landsat 8 satellite (Band 4 and Band 5) during the spring season using the website https://earthexplorer.usgs.gov/; accessed on 15 April 2021. Due to the position of the satellite in its orbit, haze, dust, and sun angle, satellite images have some defects which should be processed. Satellite images should be geometrically, atmospherically, and radiometrically corrected to become ready for extracting the required data. For that, satellite image processing software ENVI 5.3 and Arc GIS 10.5 were used to correct the collected satellite imageries. Soil Adjusted Vegetation Index (SAVI) was calculated according to the following equation:

SAVI = ((Band 5 − Band 4)/(Band 5 + Band 4 + L)) × (1 + L)).

where, L = The amount of green vegetation cover (For example, 0.5).

Then the study area (Hagul) was extracted from the satellite images.

## 3. Results

### 3.1. Floristic Composition

**Figure 3:**
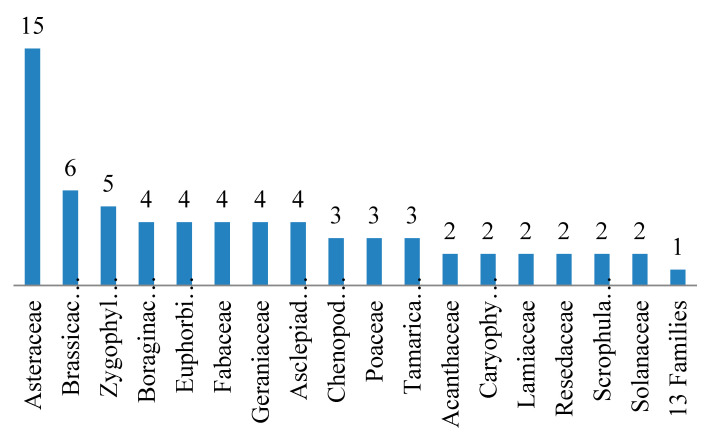
Number of species distributed across different families recorded in Wadi Hagul.

A collection of 80 plant taxa, belonging to 30 families, was recorded in Wadi Hagul. The most represented plant families were Asteraceae, Brassicaceae, and *Zygophyllaceae* with 15 (18.8%), 6 (7.5%), and 5 species (6.3%), respectively. Thirteen families (Amaranthaceae, Apiaceae, Capparaceae, Convolvulaceae, Cucurbitaceae, Ephedraceae, Malvaceae, Nitrariaceae, Orobanchaceae, Fabaceae, Plantaginaceae, Polygonaceae, and Rutaceae) were represented by one species (Table 2 and Figure 3).

**Figure 4:**
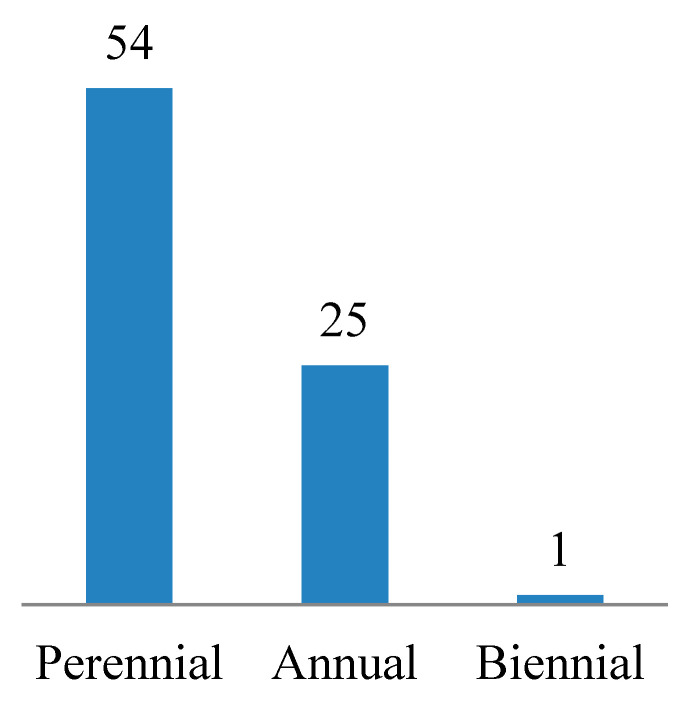
Life span of the species recorded in Wadi Hagul.

**Figure 5:**
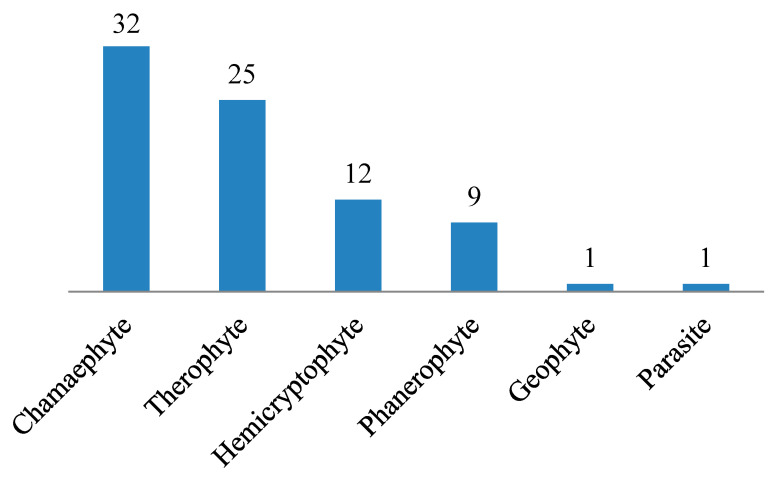
Life forms of species recorded in Wadi Hagul.

Regarding the life span of the listed species, most of them are perennials (54 species, 67.5%), followed by annuals (25 species, 31.3%). A single biennial species was *Centaurea aegyptiaca* (Table 2 and Figure 4). Comparing species according to their life form, we found approximately 40% chamaephytes, 31% therophytes, 15% hemicryptophytes, and 11% phanerophytes. Parasite and geophyte species were represented by a single species each (Table 2 and Figure 5).

**Figure 6:**
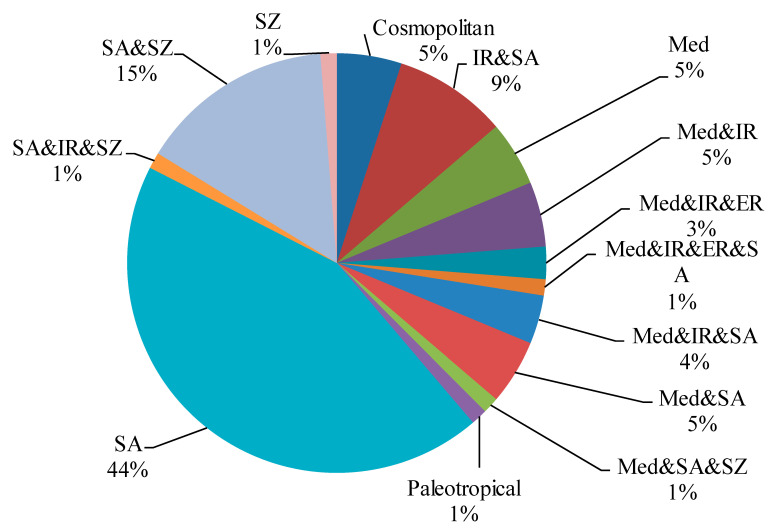
Chorotypes of the species recorded in Wadi Hagul.

With respect to a phytogeographical region, monoregional geoelements were the most common floristic category (40 species, 50%), followed by *biregionals* (27 species, 33.8%), and pleuriregionals (8 species, 10%). Cosmopolitan and palaeotropical species were represented by one and one species, respectively (Table 2 and Figure 6).

In the current study, 80 taxa were recorded, whereas Abdelaal [60] listed 98 taxa. The number of species recorded in this study was 19 (not recorded in the previous study), whereas 38 species were recorded by Abdelaal [60]. A total of 61 plant species were shared between the two studies (Table 2).

The averages of species richness, evenness, and Shannon‘s and Simpson‘s indices were 3, 0.556, 0.722, and 0.3755, respectively. Hill′s number when Q = zero, gamma, alpha and beta diversity were 63, 9.7 and 6.5, respectively. Hill′s number when Q = one, gamma, alpha and beta diversity were 8.2, 4.3 and 1.9, respectively. Hill′s number when Q = two, gamma, alpha and beta diversity were 5, 3.3 and 1.5, respectively.

### 3.2. Soil Characteristics

Soil samples were taken at a depth of 0 to 30 cm in the studied localities, and 15 soil factors were analyzed. Soil pH, TDS, and EC values ranged 7.4 to 8.5, 82 to 9840 ppm, and 0.128 to 15.375 dS/m, respectively. Calcium, magnesium, sodium, and potassium contents ranged 0.7 to 49 meq/L, 0.22 to 28 meq/L, 0.21 to 63 meq/L, and 0.104 to 11.2 meq/L, respectively. Carbonate, bicarbonate, chloride, and sulfate contents ranged between 0.15 and 0.45%, 0.305 and 0.61%, 0.5 and 43 meq/L, and 1.2 to 76 meq/L, respectively. Organic carbon, clay, and silt contents in all soil samples were less than 1.4%, 5%, and 15%, respectively. In addition, sand contribution in all soil samples was more than 81% (Table 3).

### 3.3. Effect of Environmental Factors on the Distribution of Plant Communities in the Studied Localities

**Figure 7:**
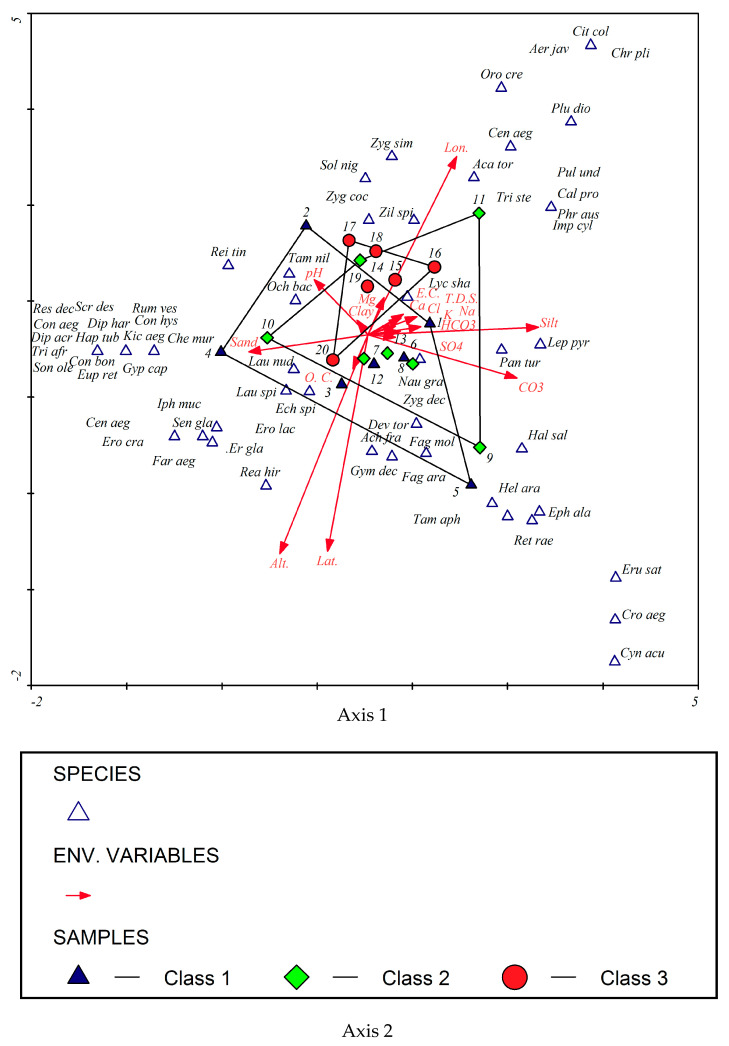
DCCA triplot showing the distribution of the studied localities and the recorded plant species as well as their relationships to environmental factors. The triangles represent species, and the arrows represent environmental variables. Arrow length expresses relative importance of an environmental variable. Dropping perpendicular to the arrows from each of the “species″ indicates the species′ relative position along the ecological gradient represented by an arrow.

The distribution of the studied localities and recorded plant species and their relationships to environmental factors are illustrated in Figure 7. DCCA showed that altitude, latitude, organic carbon content, *Tamarix aphylla, Gymnocarpos decandrus, Fagonia Arabica, Reaumuria hirtella,* and *Erodium laciniatum* positively correlated with axis 1. Longitude, Mg^2+^, clay content, *Citrullus colocynthis*, *Chrozophora plicata, Aerva javanica,* and *Orobanche crenata* are negatively correlated with axis 1. Sand content, pH, *Reseda decursiva, Scrophularia deserti, Rumex vesicarius, Conyza aegyptiaca, Diplotaxis acris, Diplotaxis harra, Haplophyllum tuberculatum, Trichodesma africanum, Sonchus oleraceus, Conyza bonariensis, Euphorbia retusa, Gypsophila capillaris, Chenopodium murale,* and *Kickxia aegyptiaca* positively correlated with axis 2, whereas T.D.S., E.C., Cl^−^, Ca^2+^, K^+^, Na^+^, SO_4_^2−^, CO_3_^−^, HCO_3_^2−^, *Pulicaria undulata*, *Calotropis procera, Phragmites australis,* and *Imperata cylindrica* negatively correlated with axis 2.

### 3.4. Vegetation Change

**Figure 8:**
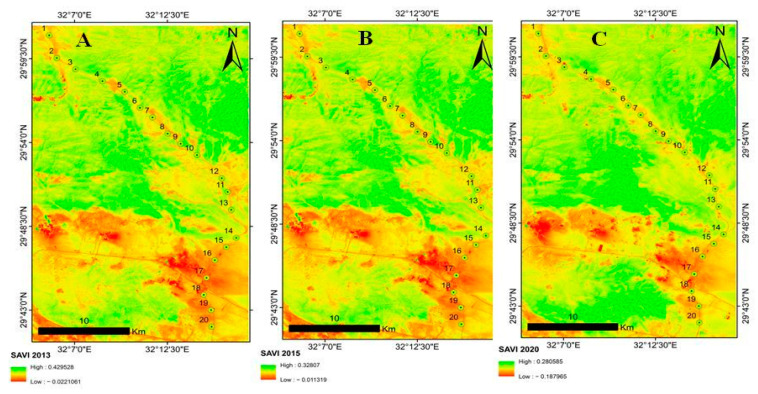
SAVI maps for 2013 (**A**), 2015 (**B**), and 2020 (**C**).

SAVI for 2013 ranged from −0.02 to 0.42; for 2015, it ranged from −0.011 to 0.32; and for 2020, it ranged from −0.18 to 0.28 (Figure 8).

## 4. Discussion

In Wadi Hagul, 80 plant species belonging to 30 families were recorded. Among them, Asteraceae, Brassicaceae, and Zygophyllaceae were the most frequent families. These results are very similar to those reported in 2009 by Zahran and Willis [44] (31 species recorded) and in 2000 by Marie [61] (37 species recorded), who found that Asteraceae and Zygophyllaceae were the most common plant families in Wadi Hagul. By contrast, in 2016, Abdelaal [60] recorded 98 species, where Asteraceae and Poaceae were the most frequent families. Eight families (Aizoaceae, Cistaceae, Cleomaceae, Liliaceae, Neuradaceae, Polygonaceae, Urticaceae and Verbenaceae), each represented by only one plant species, were recorded in Abdelaal [60], and none of them were recorded in this study. Asteraceae were reported as the most common family in other Eastern desert wadies (Wadi Asyouti and Wadi Habib) [62]. Asteraceae was known for having a proportion of salt-tolerant and xerophytic species [63]. Asteraceae makes up the bulk of floristic composition in Egypt. It is represented by 98 genera, and 234 species [48,64]. Mashaly [65] reported a list of 62 species, with no alien species found.

The perennial plant group was most represented (67.5%) in the current study; this is consistent with the results of Zahran and Willis [44], Marie [61], and Abdelaal [60], who studied vegetation in the same study area during previous years. Regarding the number of annual species in Wadi Hagul, it has changed over the years of the study. This may be due to variation in the total rainfall during the studied years.

Life forms of species depend mainly on adaptation to the environment, particularly climate [66,67,68,69]. Life forms of desert plants are closely related to precipitation [70,71] and are correlated with both landform and topography [72,73,74]. In the present study, the chamaephyte life form was most represented (40%). Therophyte was the second most common life form (31.3%). These results are in accordance with the results of Abd El-Galil [75], who studied the floristic composition of Wadi Al-Assiuty, Eastern Desert, Egypt. In arid and semi-arid regions, chamaephyte and therophyte were found to be the most common life-forms [76,77,78].

Saharo-Arabian species captured the highest percentage in the floristic categories (43.75%). This result is concordant with Zahran and Willis [44], Marie [61], and Abdelaal [60]. It is worth noting that Saharo-Arabian species are good indicators for desert environmental conditions [79,80,81].

Species richness, which refers to the number of various plant species in the stands, is one of the most important indices of species diversity. In the studied stands, the average of species richness recorded three species, which is a low number. This could be due to a variety of factors, including the severe environment and climate that characterize the study area, which may be an obstacle to the growth of some plant species. Species evenness is a description of the distribution of species abundance in a community. The average of species evenness was 0.7. Species evenness is measured on a scale of 0 to 1, with 0 representing the lowest evenness (one species has 100% coverage) and 1 representing the highest evenness (coverage is evenly spread among a number of species). This may be due to the presence of a very dominant species in a community causes the less competitive species to be suppressed [82]. Shannon index depends strongly on species richness [83]. Simpson index is not a very intuitive measure of diversity since higher values indicate lower diversity [84].

The results of DCCA analysis indicated that latitude, longitude, altitude, silt and sand contents, pH, and CO_3_^2−^ content are the most important factors affecting the distribution of vegetation in Wadi Hagul. These results are somewhat consistent with those of Mashaly [62], who stated that the most influential soil factors for the distribution of vegetation in Wadi Hagul are soil texture, Na^+^, pH, and organic matter. Abdelaal [60] mentioned that K^+^, Na^+^, organic matter, moisture content, pH, E.C., and Cl^−^ were the most affecting soil parameters for the distribution of vegetation in Wadi Hagul. Plant species associated with the increase in the proportion of sand in the soil and soil pH were *Tamarix nilotica, Ochradenus baccatus, Launea nudicalus, Launea nudicalus* and *Rumex vesicarius.* The longitude and the amount of Mg^2+^ in the soil were the most important factors associated with many plant species such as; *Zilla spinosa, Zygophyllum simplex* and *Zygophyllum coccineum*. Most of the salinity factors (E.C., T.D.S., Ca^2+^, Cl^−^ , Na^+^ and K^+^), CO_3_^2−^ , HCO_3_^−^ and the percentage of silt in soil were associated with some species such as; *Lycium shawii, Leptadenia pyrotechnica, Panicum turgidum* and *Haloxylon salicornicum.* Latitude, altitude and the amount of organic carbon in soil were important factors in the distribution of some plant species such as *Echinops spinosus, Erodium laciniatum, Erodium glaucophyllum* and *Reaumuria hirtella*.

Many of the threatened plant species recorded in previous studies were not recorded in this study, including Aizoon canariensis, Artemisia judaica, Ifloga spicata, Silene viviani, Sphaerocoma hookeri, Helianthemum lippi, Astraglus spinosus, Senna alexandrina, Salvia aegyptiaca, Schismus barbatus, Hyoscyamus muticus, and Verbena officinalis. As a result, action must be taken to safeguard threatened species by a variety of measures, including the establishment of protected areas, criminalizing exposure to endangered plants, creating a gene bank for these plants, and attempting to increase their numbers in practice.

In this study, many alien and invasive species, such as *Euphorbia prostrate*, were recorded [85]. The introduction of invasive and alien species into natural habitats represents a threat to existing species. Assaeed et al. [86] indicated that invasive and exotic plants may pose a threat to natural resources and biodiversity, especially in arid habitats. Successful invaders often exhibit great degrees of adaptability, allowing them to thrive in a variety of environments [87,88]. Plant shoot and root system features are thought to be good morphological criteria for predicting successful invasion in many habitat types [89]. Many invasive species contain allelopathic chemicals that enable them to invade and control plant communities [90].

Climate changes, in addition to human encroachments such as the construction of roads and the establishment of new cities, pose the main pressure on vegetation. Climate changes and human impact negatively affect biodiversity in several Wadies in the Egyptian Eastern Desert [91].

In this study, SAVI decreased during 2013 (from −0.02 to 0.42), 2015 (from −0.011 to 0.32), and 2020 (from −0.18 to 0.28). This result could be due to various human impacts in Wadi Hagul such as the construction of the new road Al-Galala–Hagul–Zafarana, which is 84 km long and 24 m wide and crosses Wadi Hagul, in addition to many other threats such as overgrazing, plant collection, and increasing demand for energy, which have led to exploration for oil and natural gas near Wadi Hagul. Large population growth in recent times has also led to increasing demand for building materials and opening quarries inside Wadi Hagul, which resulted in a local increase in transport and pollution. Finally, it was discovered that if environmental conditions (such as human interventions and climate changes) alter and become unsuitable for plant growth, they have a negative impact on vegetation cover, thus lowering the SAVI values.

In recent times, the preservation and protection of wildlife have become an urgent necessity, especially in the light of misuse of natural resources and encroachment of wildlife. Economic development and wildlife conservation can be simultaneously achieved by following the principles, rules, and requirements of sustainable development for balanced usage of available resources.

## 5. Conclusions

This study was conducted in an unprotected area, Wadi Hagul, Eastern desert, Egypt, to evaluate the relationship between environmental factors and the distribution of plant species, as well as evaluate the negative effects of uncontrolled human activities on both floristic composition and vegetation structure. Climate change, reflected in high temperatures and a lack of rain, also has a negative impact on vegetation cover in the study area, which is classified as a semi-arid desert. Ecosystems in general, especially deserts, are greatly affected by irresponsible human interventions. Hence, plans and strategies should be developed to conserve biodiversity. Within this context, the outcomes of this study, as well as those of other similar studies, will aid in the implementation of necessary environmental protection measures. Our study clearly showed a decreasing trend of SAVI across Wadi Hagul during 2013, 2015, and 2020. Physical factors, rather than anthropogenic, were the primary driving force for vegetation dynamics, whereas the effects of anthropogenic factors may be magnified when physical environmental factors provide unsuitable ambiance for vegetation growth. Other indexes must be measured to estimate vegetation cover, especially in unprotected areas subject to major human interventions, as can be carried out in future studies.

## Figures and Tables

**Table 1:** Global Positioning System coordinates of the studied localities in Wadi Hagul.

Stands	Latitude (N)	Longitude (E)	Altitude above Sea Level (m)	Stands	Latitude (N)	Longitude (E)	Altitude above Sea Level (m)
1	30°1′2.58345″	32°5′29.5121″	277	11	29°50′45.68″	32°16′4.86″	152
2	29°59′33.13075″	32°5′56.79499″	295	12	29°51′39.19″	32°15′43.54135″	163
3	29°58′49.0674″	32°7′3.39269″	307	13	29°49′35.08406″	32°16′17.25956″	131
4	29°58′1.74144″	32°8′38.88139″	327	14	29°47′44.49253″	32°16′35.06138″	109
5	29°57′19.91″	32°9′58.45″	286	15	29°47′7.80″	32°15′59.88″	132
6	29°56′16.55599″	32°10′52.53848″	258	16	29°46′16.54″	32°15′18.87″	113
7	29°55′38.91″	32°11′37.12″	243	17	29°45′6.97″	32°14′49.35″	89
8	29°54′34.95031″	32°12′30.83965″	223	18	29°43′59.88″	32°14′39.51″	78
9	29°53′55.03″	32°13′17.35″	213	19	29°43′0.09″	32°15′5.75″	65
10	29°53′10.26654″	32°14′15.37418″	190	20	29°41′54.08515″	32°15′7.61404″	66

**Table 2:** The list of species recorded in Wadi Hagul with their plant families, life span, life form, floristic category, and abbreviations.

Species	Family	Life Span	Life Form	Floristic Category	Record	Abbreviation
*Blepharis attenuata* Napper	Acanthaceae	Pe.	Cha.	IR and SA	B	*Ble att*
*Blepharis edulis* (Forssk.) Pers.		Pe.	Cha.	SA and SZ	B	*Ble edu*
*Aizoon canariensis* L.	Aizoaceae	An.	The.	SA and SZ	A	*Aiz can*
*Aerva javanica* (Burm.f.) Juss. ex Schult.	Amaranthaceae	Pe.	Cha.	SA and SZ	B	*Aer jav*
*Deverra tortuosa* (Desf.) DC.	Apiaceae	Pe.	Cha.	SA	A,B	*Dev tor*
*Calotropis procera* (Aiton) W.T. Aiton	Asclepiadaceae	Pe.	Pha.	SA and SZ	A,B	*Cal pro*
*Cynanchum acutum* L.		Pe.	Hem.	Med, R, and ER	A,B	*Cyn acu*
*Leptadeniapyrotechnica* (Forrsk.) Decne.		Pe.	Pha.	SA	A,B	*Lep pyr*
*Pergularia tomentosa* L.		Pe.	Cha.	SA	A,B	*Per tom*
*Achilleafragrantissima* (Forssk.) Sch. Bip.	Asteraceae	Pe.	Cha.	SA and IR	A,B	*Ach fra*
*Artemisia judaica* L.		Pe.	Cha.	SA	A	*Art jud*
*Atractylis carduus* (Forssk.) C.Chr.		Pe.	Hem.	ME and SA	A	*Atr car*
*Bidens pilosa* L.		An.	The.	PAN	A	*Bid pil*
*Centaurea aegyptiaca* L.		Bi.	The.	SA	A,B	*Cen aeg*
*Conyza aegyptiaca* (L.) Dryand.		An.	The.	Med	A,B	*Con aeg*
*Conyza bonariensis* (L.) Cronquist		An.	The.	Med	A,B	*Con bon*
*Cotula cinerea* Del.		An.	The.	SA	A	*Cot cin*
*Echinops spinosus* L.		Pe.	Hem.	Med and SA	A,B	*Ech spi*
*Ifloga spicata* (Forssk.) Sch. Bip.		An.	The.	SA	A	*Ifl spi*
*Iphiona mucronata* (Forssk.)Asch.		Pe.	Cha.	SA	A,B	*Iph muc*
*Launaea capitata* (Spreng)Dandy		Bi.	The.	SA and SZ	A	*Lau cap*
*Launea nudicalus* (L.) Hook. f.		Pe.	Hem.	SA	A,B	*Lau nud*
*Launea spinosa* (Forssk.) Sch. Bip. ex Kuntze.		Pe.	Cha.	SA	A,B	*Lau spi*
*Nauplius graveolens* (Forssk.)Wilklund		Pe.	Cha.	SA	A,B	*Nau gra*
*Pluchea dioscoridis* (L.) DC.		Pe.	Pha.	SA and SZ	A,B	*Plu dio*
*Pulicaria undulata* (L.) C.A.Mey.		Pe.	Cha.	SA nd SZ	A,B	*Pul und*
*Reichardia tingitana* (L.) Roth		An.	The.	Med and IR	B	*Rei tin*
*Senecio glaucus* L.		An.	The.	Med, IR, andSA	A,B	*Sen gla*
*Sonchus oleraceus* L.		An.	The.	COSM	A,B	*Son ole*
*Volutaria lippii* (L.) Cass. Ex Maire.		An.	The.	SA	A,B	*Vol lip*
*Anchusa humilis* (Desf.) I. M. Johnst.	Boraginaceae	An.	The.	Med and SA	B	*Anc hum*
*Heliotropiumarabinense* Fresen.		Pe.	Cha.	SA	B	*Hel ara*
*Heliotropium digynum* (Forssk.) Christens		Pe.	Cha.	SA	A,B	*Hel dig*
*Trichodesmaafricanum* (L.) R. Br.		Pe.	Cha.	SA and SZ	A,B	*Tri afr*
*Anastaticahierochuntica* L.	Brassicaceae	An.	The.	SA	A,B	*Ana hie*
*Brassica tournefortii* Gouan.		An.	The.	Med, IR, and SA	A	*Bra tou*
*Diplotaxis acris* (Forssk.) Boiss.		An.	The.	SA	A,B	*Dip acr*
*Diplotaxis harra* (Forssk.) Boiss.		Pe.	Hem.	Med and SA	A,B	*Dip har*
*Eruca sativa* Mill.		An.	The.	Med, IR, R, and SA	B	*Eru sat*
*Farsetia aegyptia* Turra.		Pe.	Cha.	SA and SZ	A,B	*Far aeg*
*Matthiola longipetala* (Vent.) DC.		An.	The.	Med and IR	A	*Mat lon*
*Zilla spinosa* (L.) Prant.		Pe.	Cha.	SA	A,B	*Zil spi*
*Cleome droserifolia* (Forssk.) Delile	Capparaceae	Pe.	Cha.	SA and IR	A,B	*Cle dro*
*Gymnocarpos decandrus* Forssk.	Caryophyllaceae	Pe.	Cha.	SA	A,B	*Gym dec*
*Gypsophila capillaris* (Forssk.) C. Chr.		Pe.	Hem.	SA and IR	A,B	*Gyp cap*
*Herniaria hemistemon* J. Gay		Pe.	Hem.	Med and SA	A	*Her hem*
*Polycarpaea repens* (Forssk.) Asch.		Pe.	Cha.	SA	A	*Pol rep*
*Silene viviani* Steud.		An.	The.	Med and SA	A	*Sil viv*
*Sphaerocoma hookeri* T. Anderson		Pe.	Cha.	SA	A	*Sph hoo*
*Anabasis setifera* Moq.	Chenopodiaceae	Pe.	Cha.	SA	B	*Ana Moq*
*Bassia indica* (Wight) Scott.		An.	The.	IR and SZ	A	*Bas ind*
*Bassia muricata* (L.) Asch.		An.	The.	IR and SA	A	*Bas mur*
*Chenopodium murale* L.		An.	The.	COSM	A,B	*Che mur*
*Haloxylonsalicornicum* (Moq.) Bunge		Pe.	Cha.	SA	A,B	*Hal sal*
*Helianthemum lippi* (L.) Dum. Cours.	Cistaceae	Pe.	Cha.	SA and SZ	A	*Hel lip*
*Cleome amblyocarpa* Barratte and Murb.	Cleomaceae	An.	The.	IR and SA	A	*Cle amb*
*Convolvulus hystrix* Vahl	Convolvulaceae	Pe.	Cha.	SA and SZ	B	*Con hys*
*Citrullus colocynthis* (L.) Schrad.	Cucurbitaceae	Pe.	Hem.	SA	B	*Cit col*
*Ephedra alata* Decne.	Ephedraceae	Pe.	Cha.	SA	B	*Eph ala*
*Chrozophora plicata* (Vahl) A. Juss. ex Spreng.	Euphorbiaceae	An.	The.	SZ	B	*Chr pli*
*Euphorbia peplus* L.		An.	The.	Med, IR, and ER	A,B	*Eup pep*
*Euphorbia prostrata* Aiton		An.	The.	PAN	A	*Eup pro*
*Euphorbia retusa* Forssk.		Pe.	Hem.	SA	A,B	*Eup ret*
*Euphorbia exigua* L.		An.	The.	Med and IR	B	*Eup*
*Acacia tortilis* (Forssk.) Hayne	Fabaceae	Pe.	Pha.	SA and SZ	A,B	*Aca tor*
*Astraglus spinosus* (Forssk.) Muschl.		Pe.	Cha.	Med, IR, and SA	A	*Ast spi*
*Crotalaria aegyptiaca* Benth.		Pe.	Cha.	SA	A,B	*Cro aeg*
*Lotus glinoides* Delile		An.	The.	SZ	A	*Lot gli*
*Melilotus indicus* (L.) All.		An.	The.	Med, IR, and SA	A	*Mel ind*
*Retama raetam* (Forssk.) Webb and Berthel.		Pe.	Pha.	SA	A,B	*Ret rae*
*Senna alexandrina* Mill.		Pe.	Cha.	SA and SZ	A	*Sen ale*
*Trigonella stellata* Forssk.		An.	The.	SA and IR	A,B	*Tri ste*
*Erodium crassifolium* L′Her.	Geraniaceae	Pe.	Hem.	SA	B	*Ero cra*
*Erodium glaucophyllum* (L.) L′Hér.		Pe.	Hem.	SA	B	*Ero gla*
*Erodium laciniatum* (Cav.) Wild. subsp. Laciniatum		An.	The.	Med	A,B	*Ero lac*
*Erodium laciniatum* (Cav.) Wild. subsp. Pulverulentum		An.	The.	Med	A,B	*Ero lac*
*Lavandula coronopifolia* Poir.	Lamiaceae	Pe.	Cha.	SA	A,B	*Lav cor*
*Salvia aegyptiaca* L.		Pe.	Cha.	IR and SA	A	*Sal aeg*
*Salvia deserti* Decne.		Pe.	Cha.	SA and IR	A,B	*Sal des*
*Asphodelus fistulosus* L.	Liliaceae	An.	The.	Med, IR, and SA	A	*Asp fis*
*Malva parvifolra* L.	Malvaceae	An.	The.	Med and IR	A,B	*Mal par*
*Neurada procumbens* L.	Neuradaceae	An.	The.	IR and SA	A	*Neu pro*
*Nitraria retusa* (Forssk.) Asch.	Nitrariaceae	Pe.	Pha.	SA	B	*Nit ret*
*Orobanche crenata* Forssk.	Orobanchaceae	An.	Parasite	Med and IR	B	*Oro cre*
*Medicago laciniata* (L.) Mill.	Fabaceae	An.	The.	SA	B	*Med (L.*
*Plantago ovata* Forssk.	Plantaginaceae	An.	The.	Med, IR, and SA	A,B	*Pla ova*
*Avena fatua* L.	Poaceae	An.	The.	PAL	A	*Ave fat*
*Imperata cylindrica* (L.) Raeusch		Pe.	Hem.	Med, IR, and SA	A,B	*Imp cyl*
*Lasiurus scindicus* Henrard.		Pe.	Hem.	SA and SZ	A	*Las sci*
*Lolium multiflorum* Lam.		An.	The.	Med, IR, and ER	A	*Lol mul*
*Panicum turgidum* Forssk.		Pe.	Hem.	SA	A,B	*Pan tur*
*Pennisetum divisum* Forssk. Ex J. F. Gmel		Pe.	Hem.	SA	A	*Pen div*
*Phalaris minor* Retz.		An.	The.	Med and IR	A	*Pha min*
*Phragmites australis* (Cav.) Trin. ex Steud.		Pe.	Geophyte	COSM	A,B	*Phr aus*
*Poa annua* L.		An.	The.	COSM	A	*Poa ann*
*Schismus barbatus* (L.) Thell.		An.	The.	Med, IR, and SA	A	*Sch bar*
*Trisetaria linearis* Forssk.		An.	The.	Med and IR	A	*Tri lin*
*Emex spinosa* (L.) Campd.	Polygonaceae	An.	The.	Med and SA	A	*Eme spi*
*Rumex vesicarius* L.	Polygonaceae	An.	The.	Med, SA, and SZ	A,B	*Rum ves*
*Caylusea hexagyna* (Forssk.) M. L. Green	Resedaceae	An.	The.	SA and SZ	A	*Cay hex*
*Ochradenus baccatus* Delile.		Pe.	Pha.	SA	A,B	*Och bac*
*Reseda decursiva* Forssk.		An.	The.	SA	A,B	*Res dec*
*Haplophyllum tuberculatum* (Forssk.) A. Juss.	Rutaceae	Pe.	Hem.	SA	A,B	*Hap tub*
*Kickxia aegyptiaca* (L.) Nabelek	Scrophulariaceae	Pe.	Cha.	Med and SA	A,B	*Kic aeg*
*Scrophularia deserti* Delile		Pe.	Cha.	SA	A,B	*Scr des*
*Hyoscyamus muticus* L.	Solanaceae	Pe.	Cha.	IR and SA	A	*Hyo mut*
*Lycium shawii* Roem. and Schult.		Pe.	Pha.	SA and SZ	A,B	*Lyc sha*
*Solanum nigrum* L.		An.	The.	COSM	B	*Sol nig*
*Reaumuria hirtella* Jaub. and Spach.	Tamaricaceae	Pe.	Cha.	IR and SA	A,B	*Rea hir*
*Tamarix aphylla* (L.) H. Karst.		Pe.	Cha.	SA, IR, and SZ	A,B	*Tam aph*
*Tamarix nilotica*(Ehrenb.) Bunge		Pe.	Pha.	SA and SZ	A,B	*Tam nil*
*Forsskaolea tenacissima* L.	Urticaceae	Pe.	Hem.	SA and SZ	A	*For ten*
*Verbena officinalis* L.	Verbenaceae	Pe.	Hem.	Med and SA	A	*Ver off*
*Fagonia arabica* L.	Zygophyllaceae	Pe.	Cha.	SA	A,B	*Fag ara*
*Fagonia mollis* Delile		Pe.	Cha.	SA	A,B	*Fag mol*
*Zygophyllumcoccineum* L.		Pe.	Cha.	SA and SZ	A,B	*Zyg coc*
*Zygophyllumdecumbens* Delile		Pe.	Cha.	SA	A,B	*Zyg dec*
*Zygophyllum simplex* L.		An.	The.	PAL	A,B	*Zyg sim*

An. = Annual, Bi. = Biennial, Pe. = Perennial, The. = Therophyte, Hem. = Hemicryptophyte, Cha. = Chamaephyte, Pha. = Phanerophyte, SA = Saharo-Sindian, Med = Mediterranean, SZ = Sudano-Zambesian, IR = Irano-Turanian, ER = Euro-Siberian, A = a species recorded only by Abdelaal [60], B = a species recorded only in the current study, and A,B = a species shared between the current study and Abdelaal [60].

**Table 3:** Descriptive statistical analysis of soil factors.

Soil Factor	Mean ± Standard Error	Range (Max–Min)	Standard Deviation	Median	Interquartile Range (IQR)
pH	8 ± 0.0736	1.1 (8.5–7.4)	0.329	8.1	0.55
T.D.S. (ppm)	1077.65 ± 508.726	9,758 (9840–82)	2,275.094	360.5	533
E.C. (dS/m)	1.684 ± 0.795	15.247 (15.375–0.128)	3.555	0.563	0.832
Ca^+2^ (meq/L)	6.353 ± 2.537	48.3 (49–0.7)	11.346	2.65	2.952
Mg^+2^ (meq/L)	3.053 ± 1.366	27.78 (28–0.22)	6.108	1.27	1.37
Na^+^ (meq/L)	6.025 ± 3.421	62.79 (63–0.21)	15.301	1.15	2.205
K^+^ (meq/L)	1.18 ± 0.565	11.096 (11.2–0.104)	2.528	0.165	1.288
CO_3_^−2^ (%)	0.225 ± 0.0231	0.3 (0.45–0.15)	0.103	0.15	0.15
HCO_3_^−^ (%)	0.32 ± 0.0153	0.305 (0.61–0.305)	0.0682	0.305	0
Cl^−^ (meq/L)	4.532 ± 2.278	42.5 (43–0.5)	10.189	1.235	1.37
SO_4_^−2^ (meq/L)	9.57 ± 3.961	74.8 (76–1.2)	17.714	3.9	4.75
Organic carbon (%)	0.888 ± 0.079	1.2 (1.32–0.12)	0.353	0.84	0.66
Sand (%)	83.333 ± 0.19	3.05 (84.7–81.65)	0.851	83.4	1.428
Silt (%)	13.31 ± 0.137	1.9 (14.3–12.4)	0.613	13.4	1.15
Clay (%)	3.357 ± 0.165	2.64 (4.7–2.06)	0.739	3.325	1.087

pH = soil reaction, T.D.S. = total dissolved salts, E.C. = electrical conductivity, Ca^2+^ = calcium, Mg^2+^ = magnesium, Na^+^ = sodium, K^+^ = potassium, CO_3_^2−^ = carbonates, HCO_3_^−^ = bicarbonates, Cl^−^ = chlorides, SO_4_^2−^ = sulfates, ppm = parts per million, dS/m = deciSiemens per meter and meq/L = milliequivalents per liter.

## Data Availability

Relevant data applicable to this research are within the paper.

## Supplementary Materials

The following are available online at https://www.mdpi.com/article/10.3390/plants10091906/s1, Figure S1: Climate diagram of Wadi Hagul (1990–2020): mean maximum temperature (°C), mean minimum temperature (°C), precipitation (mm), hot days (°C), and cold nights, Figure S2: Wind direction and velocity in Wadi Hagul (1990–2020).
