# Supplementary material for: Impacts of Anthropogenic Disturbance on Vegetation Dynamics: A Case Study of Wadi Hagul, Eastern Desert, Egypt
Source: Plants (Basel). 2021 Sep 14;10(9):1906. doi: 10.3390/plants10091906 (PMC8466335; doi:10.3390/plants10091906)
Supplement: Supplementary file 1 [file plants-10-01906-s001.zip › plants-1309866-supplementary.pdf]

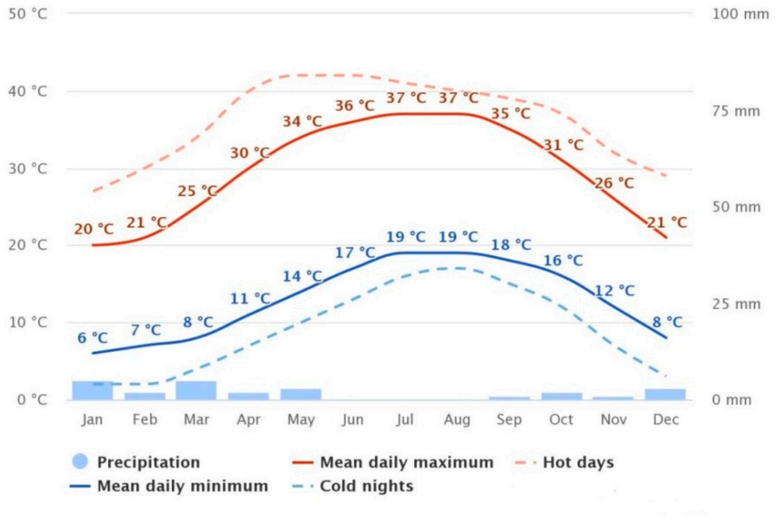

Figure S1. Climate diagram of Wadi Hagul (1990–2020): mean maximum temperature (°C), mean minimum temperature (°C), precipitation (mm), hot days (°C), and cold nights.

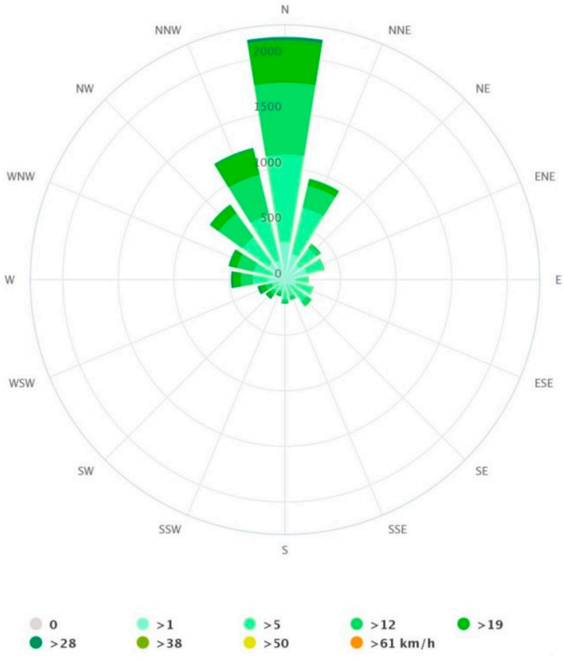

Figure S2. Wind direction and velocity in Wadi Hagul (1990–2020).
